# Orthodontic bracket bonding techniques and adhesion failures: A systematic review and meta-analysis

**DOI:** 10.4317/jced.59768

**Published:** 2022-09-01

**Authors:** André-Luiz-Campos dos Santos, Letícia-Maira Wambier, Denise-Stadler Wambier, Kelly-Maria-Silva Moreira, José-Carlos-Pettorossi Imparato, Ana-Cláudia-Rodrigues Chibinski

**Affiliations:** 1 Departament of Dentistry; State University of Ponta Grossa; Ponta Grossa, Paraná, Brazil; 2 Departament of Dentistry; São Leopoldo Mandic Faculty, Campinas, São Paulo, Brazil

## Abstract

**Background:**

This systematic review compared the bonding failures of orthodontic brackets bonded by indirect or direct techniques. Data sources: The searched databases were Cochrane Library, LILACS, BBO, PubMed, Scopus, Web of Science.

**Material and Methods:**

A search for randomized clinical trials comparing the two techniques was carried out to answer the research question: When considering orthodontic bracket bonding on permanent teeth, does the indirect technique reduce the number of bonding failures compared to the direct one over time? The quality of the included papers was assessed with Cochrane risk of bias tool and the quality of evidence with GRADE.

**Results:**

From 3096 articles identified, seven were included in the systematic review (five at unclear; two at low risk of bias). Meta-analysis was carried out according to the follow-up periods (0-6 months and 12-15 months).

**Results:**

In the first period, bonding techniques were similar with regard to adhesion failures (RR = 0.59; 95% CI 0.10-3.62; *p* = 0.00001; I2 = 92%); in the 12-to-15-month period, the direct bonding technique proved to be superior (RR = 1.44; 95% CI 1.05 - 1.99; *p* = 0.41; I2 = 0%). The quality of evidence was classified as low for the 0-6 months follow-up and high for the 12 months.

**Conclusions:**

Based on the absence of heterogeneity and the high quality of evidence, it is concluded that the direct bracket bonding technique has a lower failure rate than the indirect technique in the long term (12-15 months).

** Key words:**Orthodontic brackets, fixed orthodontics, systematic review.

## Introduction

The ideal orthodontic treatment should achieve the expected outcome, within appropriate time length and a suitable number of appointments (1). Therefore, it is essential that the brackets remain bonded to the teeth throughout the entire treatment. Failures related to the bonding of the orthodontic accessories have a prevalence of 3.5% to 10% (2-4) and they can extend treatment time, generating direct and indirect additional costs and patient dissatisfaction (5,6).

Direct bonding (DB) is used worldwide as a standard technique for attaching fixed appliances (7). However, this technique has flaws, which are inherent to the manual dexterity and clinical experience of the operators, as well as their tiredness and stress throughout the day (8,9).

To reduce these inconsistencies, an alternative technique has been gradually incorporated into the orthodontists’ practice (10,11): the indirect bracket bonding technique (IB). This technique includes clinical and laboratory steps: (a) clinical stage I - the patient’s dental arches models are obtained; (b) laboratory stage - the vertical and horizontal positioning parameters of the orthodontic accessories are defined, the accessories are fixed to the models and a transfer tray is made and (c) clinical stage II - the accessories are transferred and bonded to the teeth (12).

There are advantages and disadvantages associated with IB. For the professional, this technique allows a better visualization and greater accuracy for bracket bonding placement (13); for the patient, it provides reduced chair time (7,14). On the other hand, since indirect bonding requires laboratory procedures, it becomes more expensive (12). In addition, the bracket transfer to the mouth may result in an excessive thickness of orthodontic resin under the brackets (15) that could interfere with their position (16,17), resulting in inadequate leveling and alignment and increased treatment time (18).

Currently, with the advance of new technologies such as the computer-aided design and computer-aided manufacturing technology (CAD-CAM), renewed interest has been directed towards IB, since it allows the production of 3-dimensional (3D) modeling of the maxilla and the mandible and a rapid production of prototype transfer jigs to transfer the brackets with individualized custom resin bases (10). Notwithstanding, IB with or without the use of CAD-CAM can only be considered an option if its performance surpasses the one from DB.

In the dental literature, the clinical choice between the techniques is not an easy task. Some studies demonstrate that DB is more efficient than IB (19,20) or that IB has better performance (21) when bracket adhesion failures are considered. On the other hand, some studies report no differences between the two techniques (22-25).

Given the above, it is clear that there is still no consensus. Therefore, considering orthodontic bracket bonding techniques on permanent teeth, this systematic review and meta-analysis aimed to verify if the indirect technique reduces the number of bonding failures compared to the direct one over time.

## Material and Methods

The recommendations of the Preferred Reporting Items for Systematic Reviews and Meta-Analyses (PRISMA) were followed to report this study (26).

-Protocol and registration

This systematic review and meta-analysis was registered at the Prospective Register of Systematic Reviews (PROSPERO) under the number CRD42017078670.

-Information sources and search strategy

Controlled vocabulary (MeSH terms) and free text were combined to develop a search strategy based on the following research question:

• Population (P): patients with permanent teeth undergoing orthodontic treatment using metal brackets without age restrictions 

• Intervention (I): indirect bonding of orthodontic brackets

• Comparison (C): direct bonding of orthodontic brackets

• Outcome (O): bonding failures of orthodontic brackets

• Study design (S): randomized clinical trials

The search strategy was developed initially for PubMed. The boolean operator OR was used to combine the terms from PICO strategy; the operator AND were used to combine the different PICO components (population, intervention, and comparison). The Pubmed strategy was adapted to other databases (Latin American and Caribbean Literature on Health Sciences LILACS, Brazilian Dental Library - BBO, Cochrane Library, Scopus, Web of Science), following the truncation indicated for each base (Table 1). There was no restriction regarding the publication date or language. Gray literature was also searched through Google Scholar.

**Table 1:**
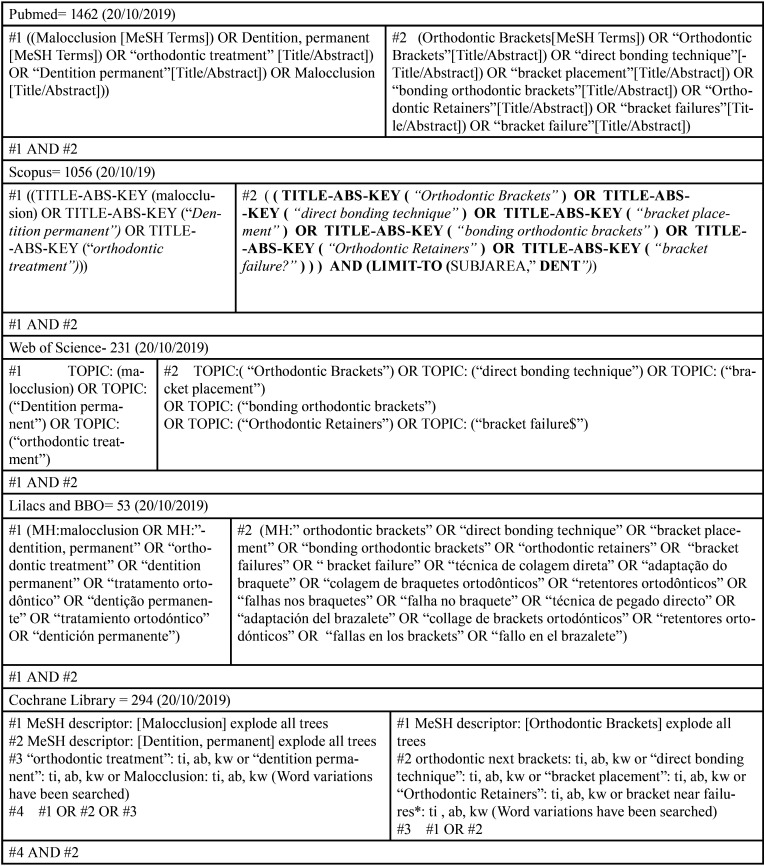
Search strategies developed for literature search in the different databases.

-Eligibility criteria

In this systematic review, randomized clinical trials (RCTs) with a parallel or split-mouth design were included, if they compare the bonding failures of orthodontic brackets in permanent teeth bonded using DB and IB. Uncontrolled clinical trials, editorial letters, historical analyses, *in vitro* studies, and case reports were excluded.

-Selection of studies and data collection process

The articles obtained were imported into EndNote X6 reference management software (Thomson Reuters, New York, NY, USA). After removing duplicates, the titles and abstracts were screened and ineligible studies were removed. This process was carried out by three reviewers (A.L.C.S, A.C.R.C. and L.M.W.).

Full texts of the remaining papers were obtained and fundamental data for the systematic review (number of participants, number of bonded brackets according to each technique and results obtained), were extracted and annotated in personalized forms. This work was carried out by three authors (A.L.C.S, A.C.R.C. and L.M.W.). In the case of reports from the same research with two different follow-up periods, the data were extracted directly to a single data record form, avoiding overlap. All these processes were conducted from February 2019 to February 2021.

-Risk of individual study bias

The Cochrane Collaboration Risk of Bias tool was used to analyze the risk of bias in randomized clinical trials (27) (Cochrane Handbook for Systematic Reviews of Interventions 5.1.0; http://handbook.cochrane.org).

There are six domains for evaluation: generation of sequence generation, allocation concealment, blinding, incomplete outcome data, selective reporting of outcomes and other possible sources of bias. In this study, two key domains were considered – sequence generation and allocation concealment - for classifying the study according to the risk of bias. As for blinding, it was not considered a key domain, since the two bracket bonding techniques have a very different protocol, making the blinding of the operator and patients unfeasible; blinding would be possible only at the time of assessment.

The risk of bias in each domain was judged to be “low”, “unclear” and “high” according to the criteria set out in the manual. Concerning each paper, it was considered to be “low risk of bias” if the two key domains (sequence generation and allocation concealment) were classified as “low” risk. If one of the key domains was judged to be of “unclear” or “high” risk, the study was considered to be of “unclear” or “high” risk of bias, respectively. Quality assessments of the included trials were performed by three independent reviewers (A.L.C.S, A.C.R.C. and L.M.W.). During the evaluation of the quality of the papers, any disagreement was resolved through discussion between the reviewers.

-Summary of measures and summary of the results

Since the data related to the outcome “failure of brackets adhesion” are dichotomous, the meta-analysis was performed to obtain an overall estimate of the risk ratio (RR), using the inverse variance method and random-effects model, with 95% confidence interval (CI). Heterogeneity was assessed using Cochran’s Q test and I2 test (inconsistency index). All analyses were performed using the Review Manager 5.3 software (Review Manager Version 5, Copenhagen, Denmark).

Studies classified as low risk or undefined risk of bias were included in the meta-analysis. Since there were different follow-up periods in the studies included, a subgroup analysis was performed, considering the follow-up periods from zero to six and from 12 to 15 months.

-Evaluation of the quality of evidence using GRADE

The quality of the evidence was assessed using the Grading of Recommendations: Assessment, Development and Evaluation (GRADE) (http://www.gradeworkinggroup.org/), aiming to identify the strength of the evidence for the outcome “bracket bond failure”.

The quality of the evidence can be classified as high, moderate, low and very low. When classified as “high quality of evidence”, it is stated that there is a high degree of confidence that the true effect is close to the estimate reported in the study (28). The quality of the evidence can be downgraded by one or two levels based on risk of bias, imprecision, inconsistency, indirect evidence and publication bias. Each criterion can be assessed as having no limitations (no downgrade); severe limitations (downgrade by 1 level); very serious limitations (downgrade by 2 levels).

## Results

**Figure 1:**
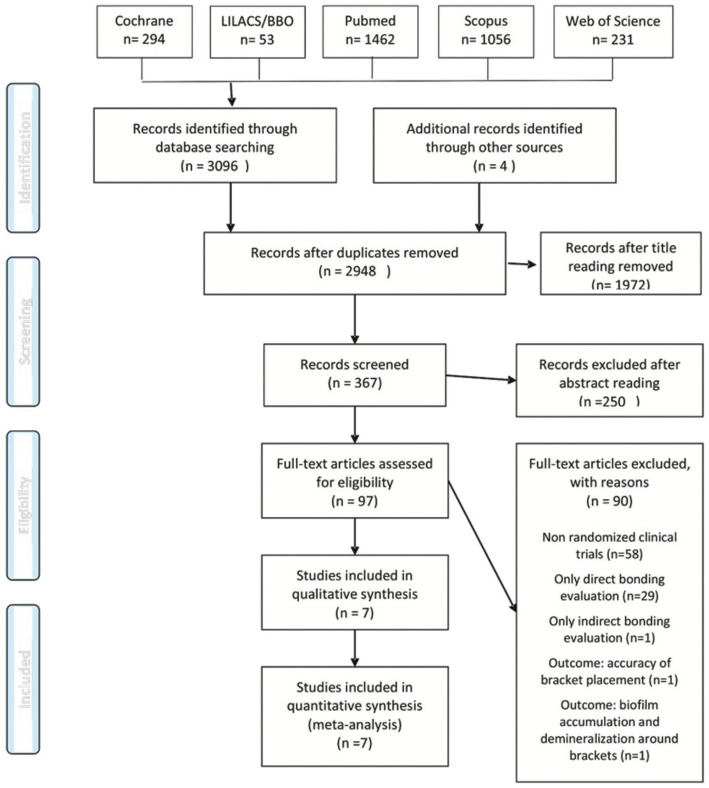
PRISMA flowchart.

A total of 3104 papers were retrieved. After removing duplicates, 2948 articles remained for the title evaluation. This phase reduced the number of papers to 367 articles. After abstracts reading, 97 studies remained according to the eligibility criteria. After full-text reading, 90 were excluded because: 1) they were not randomized clinical trials (n=58), 2) only direct bracket bonding technique was evaluated (n=5), 3) only indirect technique was evaluated (n=1), 4) failures in bracket bonding was not evaluated (n=1). Therefore, 7 studies were included in qualitative and quantitative analysis (Fig. 1) (19-25).

-Characteristics of the included studies 

The characteristics of the included studies are described in Table 2,  Table 2 cont.

**Table 2:**
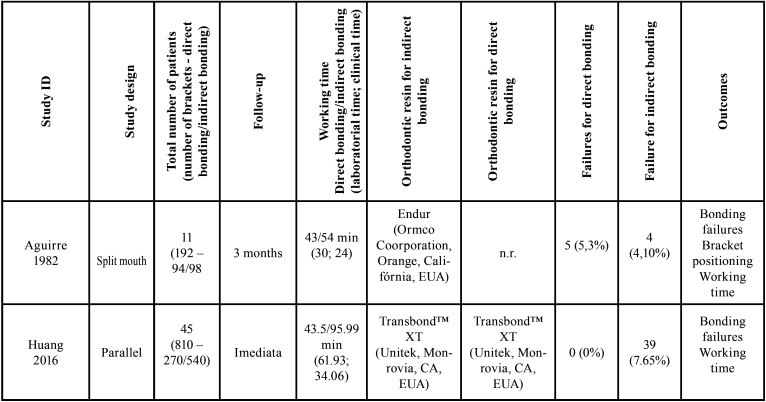
Characteristics of randomized controlled trials included in this systematic review (n=7).

**Table 2 cont.**
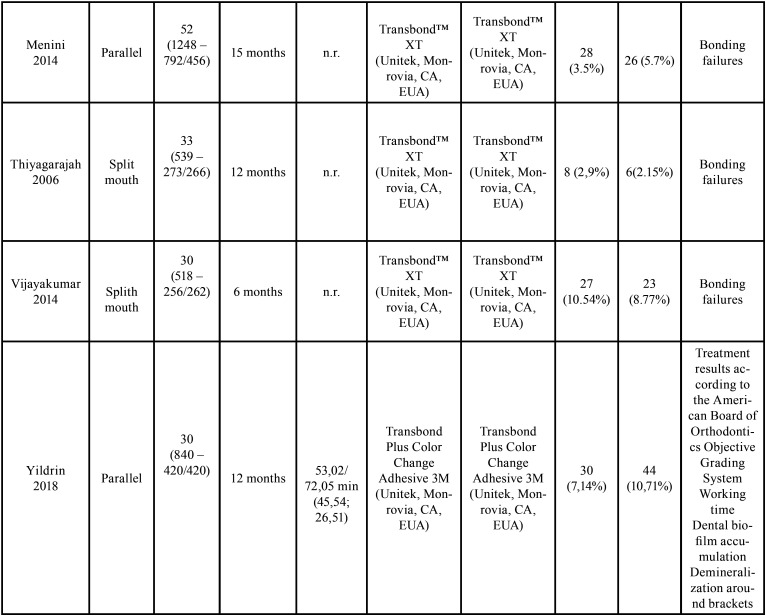
Characteristics of randomized controlled trials included in this systematic review (n=7).

All the studies used metal brackets that were bonded to the buccal face of permanent teeth and they were randomized clinical trials with split-mouth (20,21,24,25) or parallel study design (22,23,29).

The number of participants ranged from 11 to 52, in a total of 199 patients, being 70 males and 129 females. Two studies did not report the gender of the patients (24,25). The number of bonded brackets was 4591 in both techniques: 2348 for DB and 2243 for IB.

Four of the seven studies used Transbond XT ™ resin (3M, Monrovia, CA, USA) for both bonding techniques (19,22,25,30); one study used Transbond ™ Plus Color Change Adhesive (3M Unitek, Monrovia, CA, USA)(23) and two studies used the self-curing resin Endur (Ormco Corporation, Glendora California) with Concise Enamel Bonding Composite System (3M Company, St. Paul, Minnesota, USA) (20,24) . One study did not report the orthodontic resin used for IB (24).

Only three studies reported the clinical time needed for bracket bonding(19,23,24). For DB, the average time ranged from 43 to 53 minutes and for IB, it was from 24 to 34.06 minutes. The laboratory time was recorded only for IB and ranged from 30 to 61.93 minutes.

-Determination of the risk of bias

**Figure 2:**
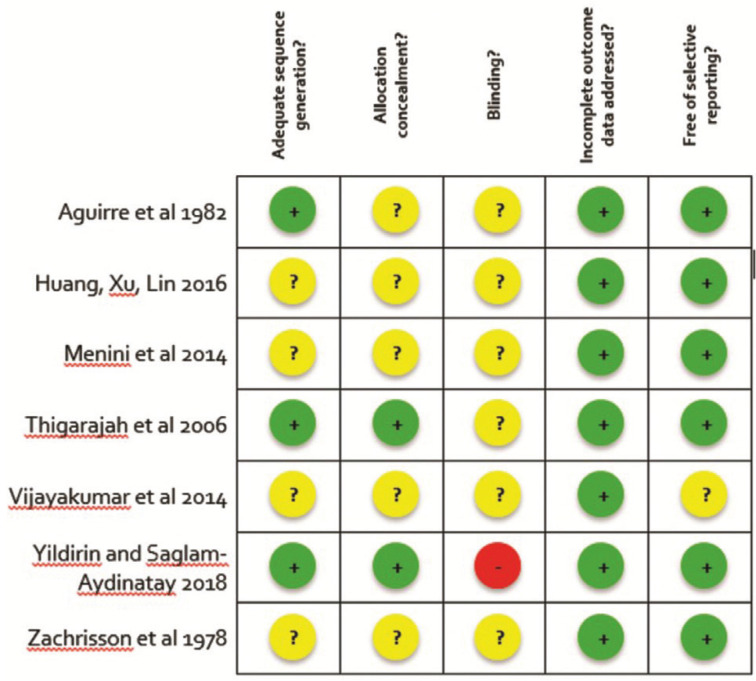
Summary of the risk of bias from randomized controlled trials included in the systematic review.

Among the seven included studies, five were considered to be at unclear (19-22,24) and two were classified as low risk of bias (23,25). No study was classified as high risk of bias, considering the key domains selected for this systematic review (sequence generation and allocation concealment). The summary of the risk of bias assessment is shown in Figure 2.

-Meta-analysis

The meta-analysis was conducted for the outcome “brackets adhesive failures” using all the studies in the systematic review (19-25).

The studies had different follow-up periods, therefore two analyses were made: short-term - 0 to 6 months (19-21,24) and long-term follow-up 12 to 15 months (22,23,25).

The short-term follow-up meta-analysis included four studies (19,20,24,30) and showed no difference between the two bracket bonding techniques concerning adhesion failures. The relative risk (RR) obtained was 0.59 (CI 95% = 0.10−3.62). The heterogeneity was considered high (I2= 92%).

**Figure 3:**
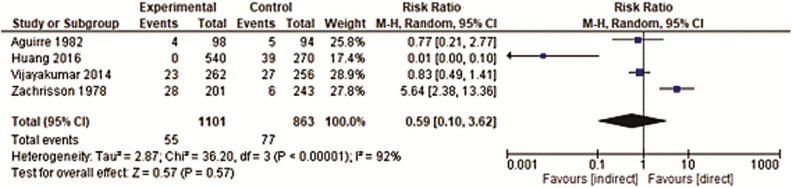
Forest-plot of short-term bracket bonding failures (0 to 6 months).

**Figure 4:**
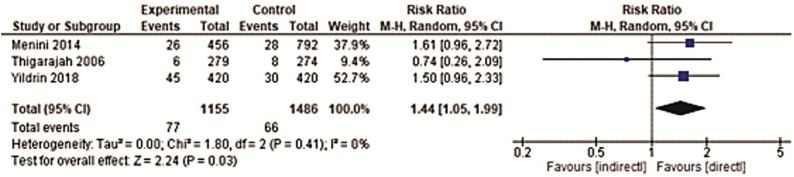
Forest-plot of long-term bracket bonding failures (12 to 15 months).

In the meta-analysis for the long-term follow-up, three studies were included (22,23,25) and the direct bonding technique showed decreased bonding failures when compared to the indirect one, with a relative risk (RR) of 1.44 (CI 95% = 1.05−1.99). There was no heterogeneity for this meta-analysis (I2=0%) (Figs. 3,4).

-Quality of evidence - GRADE

The summary of the quality of the evidence related to bracket bonding failures in the short and long-term follow-up periods is shown in Table 3.

**Table 3:**
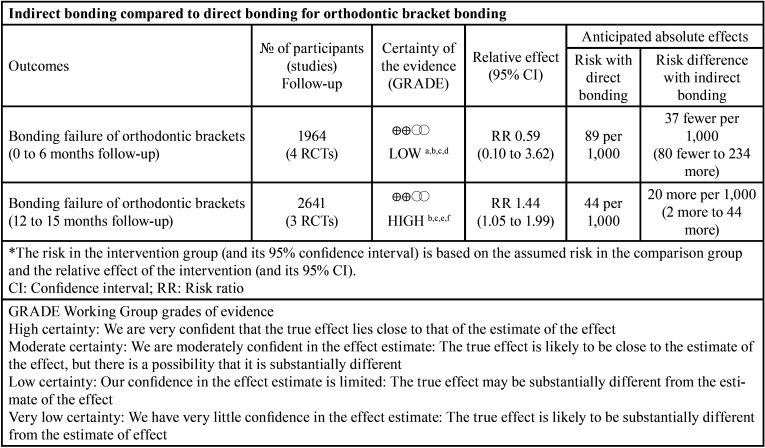
Characteristics of randomized controlled trials included in this systematic review (n=7).

In the short-term follow-up, the quality of the evidence was considered to be “low”, since confidence in the estimated effect is limited and further research may modify the conclusion obtained. The quality of the evidence was decreased due to the risk of bias (most of the data obtained comes from studies with unclear risk of bias) and imprecision (the confidence interval is very wide).

In the long-term follow-up, the quality of the evidence was considered “high”; there is confidence that the true effect is close to the estimated effect. In this follow-up period, there was no decrease in any of the evaluated criteria.

## Discussion

The adhesion of brackets to teeth is one of the factors directly related to the efficiency and duration of the orthodontic treatment (31). Therefore, the study of factors capable of minimizing such failures becomes of clinical significance for both the professional and the patient.

In this systematic review and meta-analysis, it was found that the DB has a lower adhesive failure rate compared to IB for bonding orthodontic brackets. This conclusion is based on the 12-15 months follow-up results, which showed no heterogeneity and high quality of evidence. In the period of 0-6 months, included studies were classified as unclear risk of bias (19-21,24), because the authors did not describe fundamental steps for the development of a randomized clinical trial. In the longest follow-up period, there are two articles at low risk of bias (23,25), and one at unclear risk of bias (22).

Despite the low number of studies included, it is necessary to highlight that, in the period from 12 to 15 months, the total of brackets was 2641 in DB and IB, a much higher number than the 1964 brackets bonded in the short follow-up period.

It should also be observed the narrow limit of the confidence interval for the period of 12-15 months (RR = 1.44; 95% CI = 1.05-1.99; I2=0%) when compared to the period of 0-6 months (RR = 0.59; 95% CI = 0.10-3.62; I2=92%). Therefore, it is possible to state that DB has a 44% greater chance of success compared to IB, with confidence that this result represents the true effect of the intervention.

An important question to discuss is whether the 6-month follow-up period is a clinically valid variable when it is intended to measure the effectiveness of bracket adhesion, considering that orthodontic treatment lasts, on average, 19.9 months (32).

Adhesion failures usually occur in the first 90 to 180 days after bonding the accessories (31,33). Failures occur for different reasons: bonding procedure itself (5), patient’s lack of experience with the new device, extreme occlusal forces (34). One must also consider the inherent changes in the orthodontic appliance when it is exposed to the oral environment, such as the fatigue effect related to chewing and the “aging” of the cement (31). There is a tendency to increase the number of bracket debonding as the treatment time progresses (35). In this regard, the present systematic review reinforces the advantages of using DB as a measure to minimize the need for bracket replacement, saving the clinical time of the professional and the repeated chair time of the patient.

The result of this meta-analysis may be explained by the fact that, although the positioning of the brackets is done in the laboratory, IB ends up with increasing the number of bonding phases and errors in any of these phases can lead to undesirable effects, leading to lower bond strength and failures over time (36).

Another issue is that it is not possible to guarantee that the thickness of the adhesive used in the laboratory to position the bracket is reproduced in the mouth. Excessive and irregular layers of adhesives produce low bonding resistance and failures (20,21,23,24). This situation can occur even with the use of digital flow, which is also subject to interference from the oral structures and the operator’s ability to transfer the brackets to the mouth (37).

It can also be argued that, in DB, the professional has greater control over the procedure. Since the visualization of the adhesion process is direct, without the transfer tray, if the professional identifies contamination by saliva, for example, it is possible to resume the adhesion protocol and revert the contamination, which is not possible in IB (20).

The kind of orthodontic resin used is another topic that must be discussed. In the present systematic review, of the seven studies analyzed, five (19,21-23,25) used the Transbond XT (3M Unitek, Monrovia, CA, USA) for both techniques. This is a direct-bonding light-curing resin, with a high content of inorganic fillers, suitable for bonding ceramic and metallic brackets and considered as the “gold standard” of orthodontic adhesives (38-40).

There are orthodontic resins developed specifically for IB, with low viscosity, nanometric filler particles and modified properties that allow the resin to flow under pressure, yet holding its shape after placement until light-cured, without draining around the bracket during bonding procedure (41,42). Transbond Supreme LV (3M Unitek, Monrovia, CA, USA) and Sondhi™ Rapid-Set Indirect Bonding Adhesive (3M Unitek, Monrovia, CA, USA) are some examples. However, the use of a specific resin resulted in absence of differences in the adhesion failures between DB and IB in a clinical trial (Transbond Supreme LV) (43).

Another point to be discussed is the influence of the installed malocclusion pattern. In the studies included in this systematic review, the types of malocclusion included in the sample were not described (20,22,25); only one paper stated that the sample consisted of patients with Class I occlusion and severe crowding (23).

An epidemiological study shows that there is a tendency for patients with deep overbites to have higher rates of bracket debonding. However, there are no differences observed according to the types of malocclusion (Class I, II or III), side of the arch (right or left) or arch (upper or lower) (44). Unfortunately, the available data retrieved from the studies included in this systematic review are insufficient to draw any conclusions on this topic.

It is important to say that different outcomes, besides the failure rate of bonding brackets, are presented in the studies, such as the accuracy of brackets placement, the biofilm index around the brackets and the white spot lesions development. However, these outcomes are presented in a very heterogeneous way, which makes it impossible to compare studies and perform a meta-analysis.

One limitation of this study is that the unit analysis used in the metanalysis was the bracket, when the ideal would be the patient/participant. However, considering that systematics reviews are secondary studies, we were limited to use the data provided by the primary studies. We suggest that new studies about this subject report their data showing not only the number of brackets but also the number of participants with bonding failures.

 The authors of this paper are aware of a recently published systematic review and meta-analysis on the subject (45). In the review by Li *et al*., the conclusion is that there is no difference between bracket bonding techniques regarding failures of adhesion, and the authors cited the weak evidence and the need for further randomized studies.

The main difference between our study and the previous systematic review and meta-analysis (45) is that we analyzed the data according to the periods of follow-up. The differentiated approach in the way of evaluating the available data certainly influenced the results obtained and enabled the conclusion favoring the direct technique. Also, the review by Li *et al*. (45) included only 5 studies in the meta-analysis, with no distinction between follow-up periods. In the present study, in addition to considering the follow-up period of bracket bonding failures, we used two additional articles in the meta-analysis (19,22), which increased the final sample of brackets bonded with both techniques.

An important question to discuss is whether the 6-month follow-up period is a clinically valid variable when it is intended to measure the effectiveness of bracket adhesion, considering that orthodontic treatment lasts, on average, 19.9 months (mean treatment length ranges from 14 to 33 months) (32).

Adhesion failures usually occur in the first 90 to 180 days after bonding the accessories (31,33). However, failures occur for different reasons, such as the bonding procedure itself (5), the patient’s lack of experience with the new device or extreme occlusal forces (34). One must also consider the inherent changes in the orthodontic appliance when it is exposed to the oral environment, such as the fatigue effect related to chewing, the “aging” of the cement due to changes in temperature, pH, exposure to saliva and oral enzymes (31).

Thus, it is observed that there is a tendency to increase the number of bracket debonding as the treatment time progresses (35). Considering all these characteristics inherent to orthodontic treatment, it can be said that the ideal is an adequate bonding between the enamel and the accessories, without failures during the entire treatment period. In this regard, the present systematic review reinforces the advantages of using direct bracket bonding as a measure to minimize the need for bracket replacement, saving the clinical time of the professional and the repeated chair time of the patient.

For the accumulation of biofilm and the development of white spot lesions, there are reports of decreased plaque levels and white spots with the use of IB (46,47) or similar conditions between the techniques (23). In the same way, there is no consensus regarding the accuracy of brackets placement. Some studies show a similarity between DB and IB (48) or a superiority of IB (49,50). Such variables still need new randomized clinical studies to identify whether additional advantages can be related to DB or IB.

The only characteristic that is well established in the literature is the clinical time required for bonding the brackets. In this regard, IB is faster (19,23,24), but requires additional time to perform laboratory procedures, in addition to extra costs. These two factors can also influence the clinician as well as the patient when choosing the indirect technique.

## Conclusions

The direct technique has a lower failure rate than the indirect technique for bonding orthodontic brackets in the long term follow-up (12-15 months). This conclusion is based on a set of papers with absence of heterogeneity and high quality of evidence.
